# Health equity of displaced Syrians in Lebanon: Riwa Khalifeh

**DOI:** 10.1093/eurpub/ckac130.027

**Published:** 2022-10-25

**Authors:** R Khalifeh, W D'Hoore, M Dauvrin, C Saliba

**Affiliations:** 1 Public Health, UCLouvain, Brussels, Belgium; 2 Public Health, Lebanese University, Beirut, Lebanon

## Abstract

Lebanese government data indicates that the country hosts 1.5 million displaced Syrians (DS). Providing care for DS is a challenge, especially when barriers and discrimination issues arise in accessing the Lebanese health system. This study therefore aimed to understand the causes of biases, their mechanisms, their forms and their consequences on access and quality of care for DS in Lebanon. A qualitative study using in-depth semi-structured interviews was utilized. In 2021, 28 semi-structured interviews were conducted with doctors (n = 12) and nurses (n = 16). Six group interviews were conducted with DS (n = 22) in different Lebanese healthcare facilities. The recruitment of participants was based on reasoned and targeted sampling. The interviews were recorded and transcribed for later narrative content analysis. Thematic analysis was performed to identify common themes in participants’ experiences of DS in accessing Lebanese healthcare. The results showed a barrier of access to care related to transportation and financial issues. Discrimination emerged as an underlying mechanism that drives health inequity. Several factors contributed to the presence of biases in the Lebanese healthcare system. Healthcare services provided to the Syrian population may not be the best due to inequity to access the health system attributable to the discriminatory behavior of healthcare providers. The underlying causes of discrimination are due to the fragility of the Lebanese healthcare system facing a humanitarian crisis with a collapsed infrastructure torn by previous wars and current socio-political and financial problems. Global initiatives can provide the help needed for the equitable provision of health services by providing the resources necessary to address this problem. The findings of this study highlight the changes that should be performed at the micro (cultural skills) and macro (equitable distribution of resources) levels to grant quality of healthcare services for DS.

**Key messages:**

- The health equity of DS in Lebanon is influenced by the lack of resources and the socio-political situation.
- Measures should be examined to deliver equally health services for both Lebanese and Syrians.

